# Differences in the lipid profile and hormone replacement therapy use in Korean postmenopausal women: the Korea National Health and Nutrition Examination Survey (KNHANES) 2010–2012

**DOI:** 10.1007/s00404-015-3982-9

**Published:** 2015-12-19

**Authors:** Eun Young Ki, Soo Young Hur, Jong Sup Park, Kyung Do Han, Yong Gyu Park

**Affiliations:** Department of Obstetrics and Gynecology, College of Medicine, The Catholic University of Korea, Seoul, Korea; Department of Medical Life Science, The Catholic University of Korea, 222 Banpo-daero, Seocho-gu, Seoul, 137-701 Korea

**Keywords:** Hormone replacement therapy (HRT), Dyslipidemia, Menopause, Korean National Health and Nutrition Survey (KNHANES)

## Abstract

**Purpose:**

Hormonal changes after menopause can cause dyslipidemia by the cessation of endogenous estrogen. We analyzed the lipid profile of the Korean healthy menopausal women according to the use of hormone replacement therapy (HRT).

**Methods:**

Data obtained from the Korea National Health and Nutrition Examination Survey (KNHANES) 2010–2012 were analyzed. The study included 428 healthy postmenopausal women with HRT (HRT group) and 1804 healthy postmenopausal women without HRT (NHRT group).

**Results:**

After adjustment for confounding factors, total cholesterol (TC) and low-density lipoprotein (LDL) were lower in the HRT group than in the NHRT group (TC: 200.1 ± 2.0 vs. 204.9 ± 1.1, *P* = 0.04; LDL: 120.3 ± 1.0 vs. 124.5 ± 1.0 mg/ml, *P* = 0.033). Triglycerides (TG) were lower in the HRT group than in the NHRT group [106.8, (95 % CI 99.8–114.3) vs. 115.1 (95 % CI 111.8–118.5), *P* = 0.04]. Non-high-density lipoprotein (HDL) was lower in the HRT group than in the NHRT group (145.4 ± 1.9 vs. 151.2 ± 1.0 mg/ml, *P* = 0.008). Patients with HRT were lower in the LDL cholesterol level (OR 0.601, 95 % CI 0.397–0.917, *P* = 0.018), the total cholesterol to high-density lipoprotein ratio (OR 0.787, 95 % CI 0.617–0.997, *P* = 0.016), and the non-HDL level (OR 0.68, 95 % CI 0.509–0.907, *P* = 0.009).

**Conclusion:**

The results of this study suggest that the use of HRT may have a positive effect on dyslipidemia in postmenopausal women.

## Introduction

Coronary heart disease (CHD) is a fatal disease in both men and women, increasing average life span and obese people’s attention to CHD. The relative risk of CHD-related death is increased in men (2.5–4.5) compared to women at a younger age [1]. As for age-specific incidence, the number of CHD events in men equals to that in women 10 years older. The male/female mortality ratio is 5:1 for those aged 35–44 years, and 1.5:1 for those aged over 75 years [2]. Gender differences seem to delay the risk of CHD by 10 years, a time shift usually attributed to female sex hormones. It is postulated that changes in endogenous hormones during or after menopause transition explain most of the gender differences in CHD [3].

Dyslipidemia is an important risk factor for CHD, which can be corrected and prevented. It has been defined as an elevation in total cholesterol (TC), triglycerides (TG), and low-density lipoprotein cholesterol (LDL), or a reduction in high-density lipoprotein cholesterol (HDL) [4]. Non-HDL as calculated by subtracting the HDL level from the TC level, and the lipid-related ratio have been proposed to be predictive factors for CHD [5, 6].

Hormone replacement therapy (HRT) is widely used to treat menopausal symptoms and prevent osteoporosis, cardiovascular disease, and dementia [7–9]. The regimen is estrogen alone in women undergoing hysterectomy, and estrogen combined with progestin reduces endometrial hyperplasia or cancer [10].

Some studies have found that the use of HRT is associated with an improvement in the lipid profile in healthy postmenopausal women, whereas others have found no beneficial effects [11–14]. Most of the previous studies on the relationship between HRT and lipid variables have been conducted in Western in women [5, 10, 15], while few studies have been undertaken in Asian women. This cross-sectional study was based on data from the Korea National Health and Nutrition Examination Survey (KNHANES) 2010–2012 conducted by the Korea Centers for Disease Control and Prevention. The purpose of this study was to evaluate the relationship between the use of HRT and lipid metabolism in healthy postmenopausal Korean women.

## Materials and methods

### Study subjects

This study included 428 healthy postmenopausal women with HRT (HRT group) and 1804 healthy postmenopausal women without HRT (NHRT group). It was designed to evaluate nationwide health and nutrition status, and comprised a health interviews, nutritional assessment, and health examination. The NHRT group consisted of women ranging from 38 to 64 years of age, who were aged between 30 and 60 years at menopause. The HRT group consisted of women ranging from 43 to 64 years of age, who were aged between 15 and 58 years at menopause. The HRT group included women who were receiving estrogen-alone oral therapy, combined estrogen/progestogen derivatives, or non-androgenic progesterone/transdermal estrogen therapy for at least 6 months. The duration of HRT ranged from 0.5 to 37 years. In this study, we used the following questions: (1) Have you taken hormone preparations for more than 6 months? (2) How long have you taken hormone preparations? (3) At what age did you start to take hormone preparations? Menopause was defined as a menstruation-free period of at least 12 months.

Questionnaires were used to collect a detailed history, which included age at menarche and menopause, the history of operation, pregnancy, parity, the use of oral contraceptives, the use of HRT as well as the duration of HRT and life-style variables.

The survey was reviewed and approved by the Institutional Review Board of the Seoul St. Mary’s hospital, The Catholic University of Korea (KC15EISI0188). All participants provided written informed consent.

### Life-style variables and anthropometric measurements

Alcohol consumption, smoking status, and physical activity were investigated using the self-reported questionnaire. Based on the amount of alcohol consumed per day up to 1 month before the interview, subjects who had consumed ≥30 g/day of alcohol were classified as heavy drinkers [16]. Physical activity was assessed using a short form of the International Physical Activity Questionnaire modified for the Korean population [17]. Subjects who exercised moderately for over 30 min per session more than 5 times per week, or those who exercised vigorously for over 20 min per session more than 3 times per week were defined as regular physical exercisers.

Height and body weight were measured and rounded to the nearest 0.1 cm and 0.1 kg, respectively. Body mass index (BMI) was calculated using the formula: body weight (kg)/height^2^ (m^2^). Waist circumference (WC) was measured at the midpoint between the lower costal margin and the iliac crest during expiration. Blood pressure (BP) was measured in the sitting position using a standard mercury sphygmomanometer 3 times at 5-min intervals. The average of the second and third measurements was used in the analyses.

### Biochemical measurements

Blood samples were obtained in the morning following ≥8 h of fasting. The samples were properly processed, immediately refrigerated, and transported via cold storage units to the Central Testing Institute in Seoul, Korea, and analyzed within 24 h. The conventional parameters of dyslipidemia were defined according to the criteria of the National Cholesterol Education Program Adult Treatment Panel III (NCEP/ATP III) [4, 18]. Serum TC, TG, and HDL were measured by means of a Hitachi Automatic Analyzer 7600 (Hitachi, Tokyo, Japan) using enzymatic methods with commercially available kits (Daiichi, Tokyo, Japan). The LDL level was calculated using Friedewald’s formula in subjects with a TG of ≤400 mg/dl, and it was measured directly with commercially kits (Cholestest^®^ LDL; Sekisui Medical, Tokyo, Japan) when the TG level was >400 mg/dl. The NHDL level was calculated as TC minus HDL, and high NHDL levels were defined as ≥160 mg/dl [19].

Dyslipidemia was defined as the presence of ≥1 or more of the following abnormal lipid profiles. Hypercholesterolemia was defined as (1) a TC of ≥240 mg/dl in fasting blood tests, (2) the use of a lipid-lowering agent, and (3) physician-diagnosed dyslipidemia. Hypo-HDL cholesterolemia was defined as HDL <40 mg/dl. Hyper-LDL cholesterolemia was defined as (1) LDL >160 mg/dl, (2) the use of a lipid-lowering agent or (3) physician-diagnosed dyslipidemia. Hypertriglyceridemia was defined as a TG of ≥200 mg/dl. In addition, high TC to HDL ratio (TC/HDL >5), TG to HDL ratio (>3.8), and LDL to HDL ratios (>2) were considered dyslipidemia [20–22].

### Statistical analysis

All statistical analyses were performed using the SAS version 9.2 (SAS institute, Cary, NC, USA) in a manner reflecting sampling weights and providing nationally representative estimates. A *P* value of <0.05 was considered statistically significant. The independent *t* test or *χ*^2^ test was used for the assessment of differences in clinical and biochemical characteristics between the HRT and NHRT groups. Analysis of covariance (ANCOVA) was used to determine the adjusted mean ± standard error (SE) of each lipid variables according to use of HRT. Age- and multivariate-adjusted logistic regression analyses were conducted to evaluate the relationship between dyslipidemia variables according to the use of HRT, and odds ratios (ORs) and 95 % confidence intervals (CIs) were estimated. Age, BMI, alcohol consumption, smoking, exercise, hypertension, and diabetic mellitus in subjects were considered confounding factors in multivariate analyses.

## Results

Table 1 shows the baseline characteristics of the study subjects. The mean ± SEM duration of the use of HRT was 6.2 ± 0.3 years. As for anthropometric factors, BMI and WC were higher in the NHRT group than in HRT group (BMI: 24.3 ± 0.1 vs. 23.8 ± 0.2 kg/m^2^, *P* = 0.007; WC: 81.9 ± 0.3 vs. 79.5 ± 0.5 cm, *P* < 0.001). Systolic and diastolic blood pressures were also higher in the NHRT group than in the HRT group (systolic blood pressure: 123.3 ± 0.6 vs. 120.3 ± 1.1 mmHg, *P* = 0.017; diastolic blood pressure: 77.8 ± 0.3 vs. 76.0 ± 0.6 mmHg, *P* = 0.007). As for lipid variables, TC, LDL, TG, and non-HDL were lower in the HRT group than in the NHRT group [TC: 199.4 ± 2.0 vs. 205.8 ± 1.1 mg/ml, *P* = 0.007; LDL: 119.5 ± 1.8 vs. 125.1 ± 1.0 mg/ml, *P* = 0.006; TG: 105.0 (95 % CI 97.8–112.6) vs. 115.9 (95 % CI 112.2–119.7), *P* = 0.013; non-HDL: 144.0 ± 1.9 vs. 152.0 ± 1.1 mg/ml, *P* < 0.001]. The TC/HDL ratio (3.8 ± 0.1 vs. 4.0 ± 0.0, *P* = 0.009), LDL/HDL ratio (2.3 ± 0.0 vs. 2.4 ± 0.0, *P* = 0.01), and TG/HDL ratio [2.0 (95 % CI 1.8–2.1) vs. 2.2 (95 % CI 2.1–2.3), *P* = 0.019] were lower in the HRT group than in the NHRT group. The incidence of CHD and stroke is presented in Table 2. The incidence of CHD was higher in the NHRT group than in the HRT group, but it was not statistically significant (*P* = 0.11).

**Table 1:** Characteristics of the study subjects

	NHRT (*n* = 1804)	HRT (*n* = 423)	*P* value^b^
Age, years	56.2 ± 0.1	56.3 ± 0.3	0.829
BMI, kg/m^2^	24.3 ± 0.1	23.8 ± 0.2	0.007
WC, cm	81.9 ± 0.3	79.5 ± 0.5	<0.001
Systolic blood pressure, mmHg	123.3 ± 0.6	120.3 ± 1.1	0.017
Diastolic blood pressure, mmHg	77.8 ± 0.3	76 ± 0.6	0.007
Fasting glucose, mg/ml	100 ± 0.8	95.6 ± 0.9	<0.001
Total cholesterol (TC), mg/ml	205.8 ± 1.1	199.4 ± 2.0	0.007
High-density lipoprotein (HDL), mg/ml	53.9 ± 0.3	55.4 ± 0.9	0.132
Low-density lipoprotein (LDL), mg/ml	125.1 ± 1.0	119.5 ± 1.8	0.006
TC/HDL	4.0 ± 0.0	3.8 ± 0.1	0.009
LDL/HDL	2.4 ± 0.0	2.3 ± 0.0	0.01
Non-HDL	152 ± 1.1	144 ± 1.9	<0.001
Triglycerides (TG)^a^, mg/ml	115.9 (112.2, 119.7)	105.0 (97.8, 112.6)	0.013
TG/HDL^a^	2.2 (2.1, 2.3)	2.0 (1.8, 2.1)	0.019
Delivery history			0.114
None	2.9 (0.5)	4.1 (1.2)	
1–2	53.1 (1.6)	59.7 (3.0)	
3–4	39.1 (1.5)	32.8 (3.1)	
≥5	5.0 (0.5)	3.4 (1.2)	
Smoking history (ever)	5.8 (0.8)	7.8 (1.8)	0.259
Alcohol drinking (within a month)	32.7 (1.4)	33.1 (2.8)	0.897
Exerciser	17.9 (1.2)	18.8 (2.2)	0.718
Rural resident	25.7 (2.4)	18.1 (3)	0.022
Spouse	83.4 (1.1)	84.4 (2.2)	0.694
Lipid-lowering agent	10.7 (0.9)	16.3 (2.2)	0.01
Diabetes mellitus	18.7 (1.2)	12.3 (2.5)	0.048
Hypertension	17.9 (1.3)	17.8 (1.5)	0.97

Values represent means ± SEMs or proportions (SEs)

^a^Log transformation was performed to obtain *P* values, and values represent geometric means (95 % CI)

^b^Obtained by the *t* test or the *χ* ^2^ test

**Table 2:** Incidence of coronary heart disease and stroke between NHRT and HRT group

	NHRT	HRT	*P* value
CHD	3.3 (0.5)	1.4 (0.8)	0.10
Stroke	0.7 (0.2)	0.9 (0.5)	0.68
Cardiovascular disease (CHD + stroke)	4 (0.6)	2.3 (0.9)	0.18

Valuables mean percentage (SE)

*CHD* coronary heart disease

**Fig. 1:**
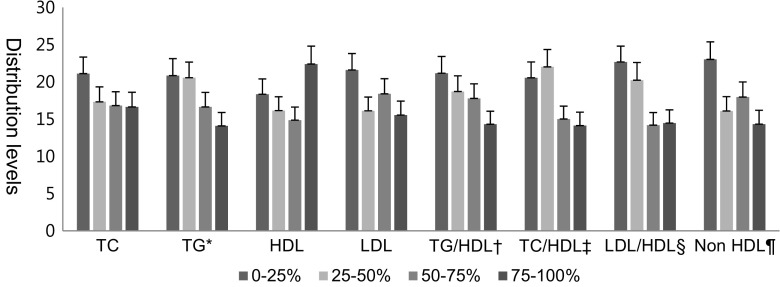
The distribution of the levels of lipid variables in quartiles in the HRT group. *P for trend 0.007, ^†^ *P* for trend 0.015, ^‡^ *P* for trend 0.003, ^§^ *P* for trend 0.001, ^¶^ *P* for trend 0.009

Figure 1 illustrates the distributions of the levels of lipid variables in quartiles in the HRT group indicating that the proportion of women with low TG levels was higher in the HRT group (*P* for trend = 0.007). In particular, such trends were noted in the TG/HD ratio (*P* for trend = 0.015), the TC/HDL ratio (*P* for trend = 0.003), the LDL/HDL ratio (*P* for trend = 0.001), and the NHDL level (*P* for trend = 0.009).

Table 3 shows changes in the levels of lipid variables according to the duration of HRT. TG, the TG/HDL ratio, Non-HDL, and the LDL/HDL ratio were proportionately decreased with increasing duration of HRT. In addition, the lowest values were noted when the duration of HRT was greater than 5 years.

**Table 3:** Time-dependent effects on lipid profile of HRT

	NHRT	0.5 year≤, >3 years	3 years≤ and >5 years	5 years≤	*P* value
TC	205.4 ± 1.1	199.2 ± 4.1	196.2 ± 4.2	200.7 ± 2.9	0.06
TG^a^	115.6 (112.2, 119)	115.7 (100.7, 132.9)	104.5 (87.6, 124.7)	103.9 (96, 112.5)	0.06
HDL	53.8 ± 0.3	54 ± 1.8	53.5 ± 1.7	55.9 ± 1.1	0.35
LDL	124.8 ± 1	118.3 ± 3.8	117.8 ± 4.2	120.6 ± 2.5	0.07
TG/HDL^a^	2.2 (2.1,2.3)	2.2 (1.9, 2.6)	2 (1.6, 2.5)	1.9 (1.7, 2.1)	0.08
TC/HDL	4.0 ± 0.0	3.9 ± 0.1	3.9 ± 0.2	3.8 ± 0.1	0.06
Non-HDL	2.4 ± 0.0	2.3 ± 0.1	2.4 ± 0.2	2.2 ± 0.1	0.02
LDL/HDL	151.6 ± 1.0	145.2 ± 4.0	142.7 ± 4.2	144.8 ± 2.6	0.01

Values represent means ± SEMs or proportions (SEs)

^a^Geometric mean (95 % CI)

Table 4 shows the mean levels of lipid variables in both groups according to the use of HRT after adjustment. In Model 3, after adjustment for age, BMI, smoking, drinking, exercise, DM, hypertension, and the use of lipid-lowering agents, TC, TG, LDL, and non-HDL were significantly lower in the HRT group than in the non-HRT group (TC: 204.9 ± 1.1 vs. 201.1 ± 2.0, *P* = 0.04; TG: 115.1 vs. 106.8 *P* = 0.04; LDL: 124.5 ± 1.0 vs. 120.3 ± 1.7, *P* = 0.033; non-HDL: 151.2 ± 1.0 vs. 145.4 ± 1.9, *P* = 0.008).

**Table 4:** Differences in the levels of lipid variables according to the use of hormonal agents

	Model 1^b^			Model 2^c^			Model 3^d^		
	NHRT	HRT	*P* value	NHRT	HRT	*P* value	NHRT	HRT	*P* value
TC	205.4 ± 1.1	199.5 ± 2.0	0.013	205.2 ± 1.1	199 ± 2.1	0.01	204.9 ± 1.1	200.1 ± 2.0	0.04
TG^a^	115.5 (112.1, 119)	107.2 (100.1, 114.8)	0.054	115.3 (111.9, 118.7)	106.5 (99.4, 114.1)	0.041	115.1 (111.8, 118.5)	106.8 (99.8, 114.3)	0.04
HDL	53.8 ± 0.3	54.9 ± 0.9	0.237	53.8 ± 0.3	54.9 ± 0.9	0.259	53.7 ± 0.3	54.7 ± 0.9	0.302
LDL	124.9 ± 1	119.4 ± 1.8	0.009	124.8 ± 1	119.2 ± 1.8	0.008	124.5 ± 1	120.3 ± 1.7	0.033
TC/HDL	4.0 ± 0.0	3.8 ± 0.1	0.03	4.0 ± 0.0	3.8 ± 0.1	0.028	4.0 ± 0.0	3.9 ± 0.1	0.089
LDL/HDL	2.4 ± 0.0	2.3 ± 0.0	0.022	2.4 ± 0.0	2.3 ± 0.0	0.022	2.4 ± 0.0	2.3 ± 0.0	0.08
Non-HDL	151.6 ± 1.0	144.5 ± 1.9	0.002	151.5 ± 1.0	144.2 ± 1.9	0.001	151.2 ± 1.0	145.4 ± 1.9	0.008
TG/HDL^a^	2.2 (2.1, 2.3)	2.0 (1.8, 2.2)	0.069	2.2 (2.1, 2.3)	2.0 (1.8, 2.2)	0.059	2.2 (2.1, 2.3)	2.0 (1.8, 2.2)	0.073

Values expressed as means ± SEMs. *P* value was obtained by ANCOVA

*NHRT* group without hormone replacement therapy, *HRT* group with hormone replacement therapy, *TC* total cholesterol, *TG* triglyceride, *HDL* high-density lipoprotein, *LDL* low-density lipoprotein, *non-HDL* non-high-density lipoprotein

^a^Values expressed as geometric means (95 % CI)

^b^Adjusted for age and body mass index (BMI)

^c^Adjusted for age, BMI, smoking, drinking, and exercise

^d^Adjusted for age, BMI, smoking, drinking, exercise, diabetes mellitus, hypertension, and medication

Table 5 presents adjusted odds ratios in the HRT and non-HRT groups according to the diagnostic criteria for dyslipidemia. In Model 3, after adjustment for age, BMI, drinking, DM, hypertension, and medication, the HRT group showed a low hyper-LDL-cholesterolemia (OR 0.601, 95 % CI 0.394–0.917, *P* = 0.018), a low TC/HDL ratio (OR 0.787, 95 % CI 0.617–0.997, *P* = 0.016), and a low non-HDL level (OR 0.68, 95 % CI 0.509–0.907, *P* = 0.009). In Model 4, after adjustment for age, BMI, drinking, DM, hypertension, medication, triglyceride, and cholesterol, the HRT group showed a low hyper-LDL cholesterolemia (OR 0.61, 95 % CI 0.4–0.92, *P* = 0.021) and a low non-HDL level (OR 0.725, 95 % CI 0.539–0.977, *P* = 0.034).

**Table 5:** Adjusted odds ratios according to the criteria for dyslipidemia in the HRT and NHRT groups

	Model 1^a^ OR (95 % CI)	*P* value	Model 2^b^ OR (95 % CI)	*P* value	Model 3^c^ OR (95 % CI)	*P* value	Model 4^d^ OR (95 % CI)	*P* value
Hypercholesterolemia	1.058 (0.778, 1.439)	0.72	1.053 (0.773, 1.434)	0.743	0.703 (0.471, 1.049)	0.085	0.747^e^ (0.493, 1.13)	0.168
Hypertriglyceridemia	0.819 (0.537, 1.25)	0.355	0.808 (0.527, 1.238)	0.33	0.832 (0.544, 1.272)	0.396	0.866^f^ (0.564, 1.311)	0.512
Hypo-HDL cholesterolemia	1.156 (0.762, 1.754)	0.495	1.161 (0.764, 1.764)	0.485	1.207 (0.789, 1.846)	0.386	1.393^e^ (0.889, 2.183)	0.148
Hyper-LDL cholesterolemia	1.002 (0.728, 1.38)	0.99	1.008 (0.732, 1.388)	0.962	0.601 (0.394, 0.917)	0.018	0.61^e^ (0.4, 0.92)	0.021
TG/HDL	0.783 (0.612, 1.002)	0.06	0.768 (0.599, 0.986)	0.057	0.778 (0.609, 1.04)	0.082	0.749^f^ (0.527, 1.066)	0.108
TC/HDL	0.74 (0.581, 0.942)	0.005	0.735 (0.577, 0.937)	0.005	0.787 (0.617, 0.997)	0.016	0.758^e^ (0.55, 1.046)	0.09
LDL/HDL	0.818 (0.623, 1.073)	0.146	0.8 (0.607, 1.055)	0.115	0.851 (0.643, 1.127)	0.261	0.933^e^ (0.694, 1.255)	0.648
Non-HDL	0.656 (0.494, 0.87)	0.004	0.652 (0.491, 0.865)	0.003	0.68 (0.509, 0.907)	0.009	0.725^e^ (0.539, 0.977)	0.034

*OR* odds ratio, *CI* confidence interval, *TC* total cholesterol, *TG* triglyceride, *HDL* high-density lipoprotein, *LDL* low-density lipoprotein, *Non-HDL* non-high-density lipoprotein

^a^Adjusted for age and body mass index (BMI)

^b^Adjusted for age, BMI, smoking, drinking, and exercise

^c^Adjusted for age, BMI, smoking drinking, exercise, diabetes mellitus, hypertension, and medication

^d^Adjusted for age, BMI, smoking, exercise, diabetes mellitus, hypertension, medication, total cholesterol and triglyceride

^e^In model 4, adjusted for triglyceride

^f^In model 4, adjusted for cholesterol

There were significant differences in the levels of lipid variables between the NHRT and HRT groups according to the use of lipid-lowering agents (Table 1, *P* = 0.01). Table 6 shows the adjusted odds ratios in the NHRT and HRT groups according to the use of lipid-lowering agents. In women with no use of lipid-lowering agents, hyper-LDL cholesterolemia (OR 0.524, 95 % CI 0.322–0.853, *P* = 0.009), the TG/HDL ratio (OR 0.671, 95 % CI 0.454–0.992, *P* = 0.04), and the non-HDL level (OR 0.71, 95 % CI 0.51–0.97, *P* = 0.03) were significantly lower in the HRT group than in the NHRT group. In women with use of lipid-lowering agents, hypercholesterolemia (OR 4.63, 95 % CI 0.86–24.87, *P* = 0.07) and hyper-LDL cholesterolemia (OR 5.33, 95 % CI 0.99–28.48, *P* = 0.05) were higher in the HRT group than in the NHRT group, but the difference was not statistically significant.

**Table 6:** Adjusted odds ratios according to use of lipid-lowering agents in the HRT and NHRT groups

No medication	Model 1^a^ OR (95 % CI)	*P* value	Model 2^b^ OR (95 % CI)	*P* value	Model 3^c^ OR (95 % CI)	*P* value	Model 4^d^ OR(95 % CI)	*P* value
Hypercholesterolemia	0.665 (0.434, 1.019)	0.06	0.654 (0.423, 1.01)	0.055	0.642 (0.413, 0.998)	0.04	0.687 (0.436, 1.084)^e^	0.10
Hypertriglyceridemia	0.808 (0.507, 1.289)	0.371	0.797 (0.497, 1.277)	0.345	0818 (0.513, 1.303)	0.397	0.86 (0.54, 1.37)^f^	0.52
Hypo-HDL cholesterolemia	1.106 (0.698, 1.753)	0.667	1.116 (0.703, 1.771)	0.640	1.137 (0.714, 1.81)	0.588	1.32 (0.80, 2.18)^e^	0.26
Hyper-LDL cholesterolemia	0.514 (0.318, 0.833)	0.006	0.518 (0.319, 0.841)	0.007	0.515 (0.315, 0.84)	0.008	0.524 (0.322, 0.853)^e^	0.009
TG/HDL	0.648 (0.439, 0.956)	0.028	0.636 (0.427, 0.947) 0.025	0.025	0.654 (0.442, 0.968)	0.03	0.671 (0.454, 0.992)^f^	0.04
TC/HDL	0.675 (0.494, 0.922)	0.013	0.6645 (0.484, 0.911)	0.011	0.661 (0.482, 0.90)	0.01	0.732 (0.517, 1.307)^e^	0.08
LDL/HDL	0.687 (0.507, 0.931)	0.015	0.677 (0.5, 0.918)	0.012	0.66 (0.48, 0.89)	0.008	0.943 (0.691, 1.288)^e^	0.71
Non-HDL	0.899 (0.676, 1.195)	0.461	0.869 (0.649, 1.165)	0.35	0.852 (0.635, 1.143)	0.28	0.71 (0.51, 0.97)^e^	0.03
With lipid-lowering medication^g^								
Hypercholesterolemia	4.183 (0.786, 22.26)	0.09	4.51 (0.85, 23.7)	0.07	4.65 (0.87, 24.8)	0.07	4.63 (0.86, 24.87)^e^	0.07
Hypertriglyceridemia	0.853 (0.327, 2.24)	0.74	0.84 (0.33, 2.21)	0.71	0.819 (0.326, 2.06)	0.67	0.80 (0.31, 2.07)^f^	0.65
Hypo-HDL cholesterolemia	4.170 (0.60, 4.79)	0.31	2.09 (0.70, 6.17)	0.18	1.996 (0.662, 6.019)		2.13 (0.74, 6.12)^e^	0.15
Hyper-LDL cholesterolemia	4.6 (0.88, 4.79)	0.07	4.99 (0.96, 25.82)	0.054	5.272 (0.995, 6.019)	0.22	5.33 (0.99, 28.48)^e^	0.05
TG/HDL	1.23 (0.56, 2.68)	0.60	1.295 (0.548, 2.872)	0.524	1.26 (0.561, 2.831)	0.57	1.27 (0.5, 2.88)^‡^	0.56
TC/HDL	0.93 (0.46,1.88)	0.85	0.932 (0.461, 1.884)	0.845	0.918 (0.455, 1.855)	0.81	0.82 (0.40, 1.68)^e^	0.60
LDL/HDL	0.79 (0.387,1.64)	0.53	0.809 (0.387, 1.64)	0.53	0.788 (0.378, 1.645)	0.52	0.74 (0.34, 1.6)^e^	0.45
Non-HDL	0.74 (0.29, 1.85)	0.52	0.74 (0.29, 1.85)	0.54	0.692 (0.268, 1.789)	0.44	0.62 (0.23, 1.69)^e^	0.35

*OR* odds ratio, *CI* confidence interval, *TC* total cholesterol, *TG* triglyceride, *HDL* high-density lipoprotein, *LDL* low-density lipoprotein, *Non-HDL* non-high-density lipoprotein

^a^Adjusted for age and body mass index (BMI)

^b^Adjusted for age, BMI, smoking, drinking, and exercise

^c^Adjusted for age, BMI, smoking drinking, exercise, diabetes mellitus, and hypertension

^d^Adjusted for age, BMI, smoking, exercise, diabetes mellitus, hypertension, total cholesterol and triglyceride

^e^In model 4, adjusted for triglyceride

^f^In model 4, adjusted for cholesterol

^g^Lipid-lowering agents were used in the NHRT group (193/1804, 10.7 %) and the HRT group (69/423, 16.3 %)

**Fig. 2:**
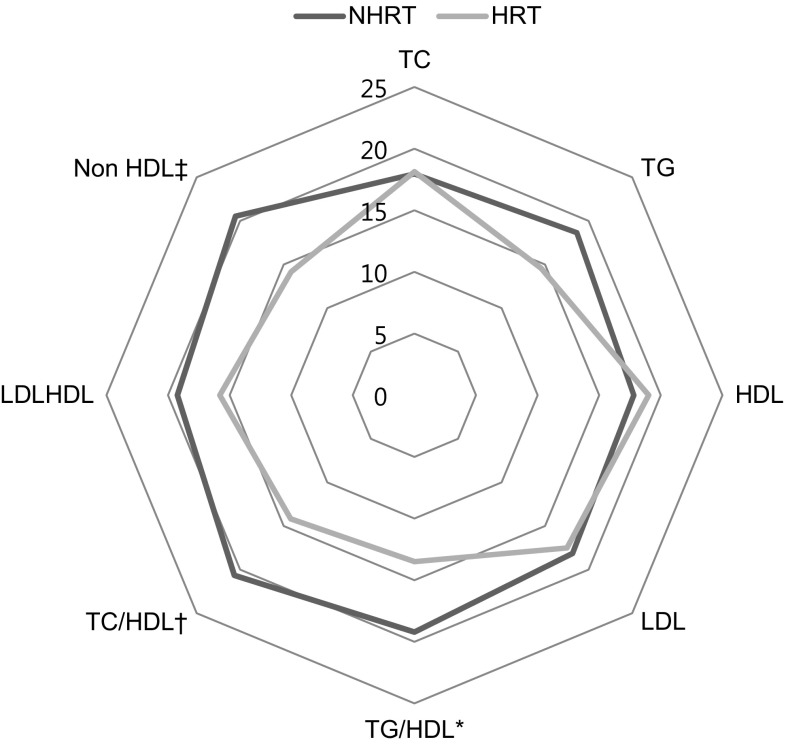
The distribution of dyslipidemia in the HRT and NHRT groups. *P = 0.018, ^†^ *P* = 0.002, ^‡^ *P* = 0.002

Figure 2 shows the distribution of dyslipidemia according to the use of HRT. It shows statistically significant differences in NHDL, the TC/HDL ratio, and the TG/HDL ratio between the 2 groups.

## Discussion

Hormone replacement therapy is widely used in menopausal women to treat menopausal vasomotor symptoms and prevent osteoporosis, cardiovascular disease, and dementia. Estrogen also affects vascular tone and blood flow as well as lipid metabolism [7, 23, 24]. Previous studies have been undertaken to determine the effects of the loss of endogenous sex hormones on lipid and lipoprotein metabolism.

The postmenopausal estrogen/progestin intervention (PEPI) Trial has indicated that HRT increases HDL, decreases LDL, but does not affect the occurrence of CHD [25]. Mathew et al. [26] have demonstrated that only total cholesterol, LDL, and apolipoprotein-B increase within the 1-year interval before and after the final menstrual period [26]. Nerbrand et al. [15] conducted the Women’s Health in the Lund Area (WHILA) study in 6908 women grouped into premenopausal women, postmenopausal women without HRT, and postmenopausal women with HRT, and examined differences in serum lipid variables between the groups. They reported that cessation of endogenous sex hormones after menopause increases TG and TC, and decreases HDL, which can be reversed by the use of HRT. Crespo et al. [5] examined the relationship of HRT with fasting glucose and lipid variables in diabetic and nondiabetic postmenopausal women. They reported that among diabetic women current users of HRT have significantly different lipid and glucose levels than never user HRT for the following variables: TC (225 vs 241 mg/dl), non-HDL (169 vs 188 mg/dl), and glucose (112 vs 154 mg/dl). Likewise, our study revealed that TC, LDL, non-HDL, and TG levels were lowered. However, the HDL level was not affected by HRT, while it was increased by HRT in several previous studies [10, 15, 27]. The use of HRT increases the HDL level, and the magnitude of increase appears to be related to the type and dosage of estrogen as well as the route of administration, but progestin has a negative effect on HDL [10].

When we analyze the hormonal effect on lipid variables, we must consider aging. Aging itself decreases HDL and increases TC, LDL, and TG [28]. Hall et al. [29] followed up 143 women for 5 years through menopause with annual measurements. They reported that transition to menopause is accompanied by increases in serum TC and TG, which is consistent with our results. Some investigators have reported the time course of changes in the lipid profile. Kika et al. [27] have demonstrated that HDL is significantly increased 12 months after the start of HRT and that at the 3 months later in HRT group, TC and LDL levels are decreased, while the TG level is increased 3 months after the start of HRT. Also Park et al. [30] have documented that 12 weeks after HRT, HDL and TG levels are increased by 12 % and TG 20 %, respectively, while LDL and lipoprotein(a) levels are decreased by 9 and 36 %, respectively, in postmenopausal patients with end-stage renal disease. Kim et al. [31] reported the effects of HRT on lipid variables in postmenopausal women according to various progestogens and duration of therapy. HDL was more increased in subjects on estrogen-alone therapy than in those on other therapies (16.5 vs. 10.8–13.8 %, *P* < 0.001), which may have been attributed to the androgenic effects of progestogens [8, 9]. A combination of estrogen and medroxyprogesterone significantly decreased the levels of LDL (17.6 vs. 10.9–13.7 %, *P* < 0.01) and TG (32.6 vs. 0–20.7 %, *P* < 0.001). The levels of lipid variables were changed in a time-dependent manner.

This study has some limitations at this point. First, this is a cross-sectional study, and thus it did not analyze the changing patterns of lipid variables according to the use of HRT. Second, since this study did not categorize hormonal agents, it did not present differences according to the different types of the agents. Since this is a cross-sectional study using a questionnaire that included the questions about the use of HRT, but the types of hormonal agents, it is difficult to evaluate the differences in the levels of lipid variables according to the types of the agents. However, this study has clinical implications in that it is the first large-scale study of Korean healthy menopausal women. Further prospective studies on the effects of HRT with various combinations of sex hormones and duration of treatment on changes in lipid variables would be warranted.
